# High-yield production of functional soluble single-domain antibodies in the cytoplasm of *Escherichia coli*

**DOI:** 10.1186/1475-2859-12-97

**Published:** 2013-10-27

**Authors:** Kristof Zarschler, Stefanie Witecy, Franz Kapplusch, Christian Foerster, Holger Stephan

**Affiliations:** 1 Institute of Radiopharmaceutical Cancer Research, Helmholtz-Zentrum Dresden-Rossendorf, Bautzner Landstraße 400, 01328 Dresden, Germany; 2 Current address: Department of Oncology, Cross Cancer Institute, University of Alberta, 11560 University Ave, Edmonton, Alberta T6G 1Z2 Canada

## Abstract

**Background:**

For their application in the area of diagnosis and therapy, single-domain antibodies (sdAbs) offer multiple advantages over conventional antibodies and fragments thereof in terms of size, stability, solubility, immunogenicity, production costs as well as tumor uptake and blood clearance. Thus, sdAbs have been identified as valuable next-generation targeting moieties for molecular imaging and drug delivery in the past years. Since these probes are much less complex than conventional antibody fragments, bacterial expression represents a facile method in order to produce sdAbs in large amounts as soluble and functional proteins.

**Results:**

By the combined use of high cell density cultivation media with a genetically engineered *E. coli* mutant strain designed for the cytoplasmic formation of proper disulfide bonds, we achieved high level of intracellular sdAb production (up to 200 mg/L). Due to a carboxyterminal hexahistidine epitope, the soluble recombinant sdAbs could be purified by one-step immobilized metal affinity chromatography to apparent homogeneity and easily radiolabeled with ^99m^Tc within 1 h. The intradomain disulfide bridge being critical for the stability and functionality of the sdAb molecule was shown to be properly formed in ~96% of the purified proteins. *In vitro* binding studies confirmed the high affinity and specificity of the expressed sdAb 7C12 towards its molecular target.

**Conclusions:**

Our study demonstrates an efficient cultivation and expression strategy for the production of substantial amounts of soluble and functional sdAbs, which may be adopted for high-yield production of other more complex proteins with multiple disulfides as well.

## Background

In a variety of solid tumors, including head and neck, breast, non-small-cell lung and pancreatic cancer, members of the human epidermal growth factor receptor family are overexpressed and/or deregulated [1-4]. The most prominent members of this family, EGFR and HER-2, represent validated targets for anticancer therapy and the current successful approaches include (i) antibodies such as Cetuximab (ImClone) and Panitumumab (Amgen) binding the extracellular ligand binding domain (ECD) as well as (ii) small molecule tyrosine kinase inhibitors (TKIs) such as Gefitinib (Astra-Zeneca) and Erlotinib (Roche) [5]. The former therapy prevents EGFR ligands from interacting and activating the receptor as well as receptor-ligand internalization, whereas the latter approach focuses on blocking adenosine triphosphate binding to the intracellular TK domain of EGFR, thereby inhibiting TK activity and subsequent intracellular signaling [6,7].

Within the last ten years, small recombinant antibody fragments have gained importance in the area of antibody-based anticancer therapies and diagnostics [8-11]. Single-domain antibodies (sdAbs), which are derived from camelid heavy chain-only antibodies and which consist solely of the antigen-specific domain [12], offer multiple advantages over conventional antibodies and fragments thereof in terms of size, stability, solubility as well as tumor uptake and blood clearance [13,14]. Several research groups described recently the construction, selection, and use of EGFR-binding sdAbs for tumor targeting, active drug delivery and radioimmunodetection of EGFR overexpressing tumors [15-18]. Both sdAbs investigated in this study, 7C12 and EG2, showed affinities to recombinant EGFR in the nanomolar range as determined by surface plasmon resonance [16,17]. Binding of 7C12 to EGFR-presenting A431 cells could be blocked *in vitro* and *in vivo* by the addition of Cetuximab [17,19], suggesting that both antibody formats bind to overlapping or adjacent epitopes on the receptor. Furthermore, Roovers and colleagues identified 7C12 as EGF antagonist that inhibits EGF-induced phosphorylation of EGFR dose dependently [20]. However, no effector function such as ligand-competitive inhibition of EGFR activation has been described for EG2.

The recombinant production of the intramolecular disulfide containing sdAbs has mainly been achieved by periplasmic and cytoplasmic expression using bacteria [21,22] or by expression and targeting to the secretory pathway of yeast [23,24]. However, methods for the production of sdAbs in transgenic plants [25], mammalian cell lines [26] and insect cells [27] have been described recently as well. Since every expression system has its advantages, limitations and drawbacks [28,29], we focus on the efficient disulfide bond formation as well as the obtainment of a high level of soluble and correctly folded product. Both issues are of special importance for economic large-scale production of sdAbs for their direct application in therapy and diagnosis or their further functionalization with nanoparticles and other surfaces [30-32].

In this study we report on the high-yield production of functional soluble single-domain antibodies in the cytoplasm of *E. coli*. Therefore, we use a genetically engineered double mutant strain defective in both, the thioredoxin (*trxB*) and glutathione (*gor*) pathways, which constitutively expresses the disulfide bond isomerase DsbC in the cytoplasm [33]. In combination with an enzyme-based glucose release system for fed-batch-like cultivation conditions, we obtain growth to higher cell densities, improved recombinant protein expression and excellent yields of up to 200 mg/L native, biologically active sdAb.

## Results

### Cloning of the sdAb open reading frames

**Figure 1:**
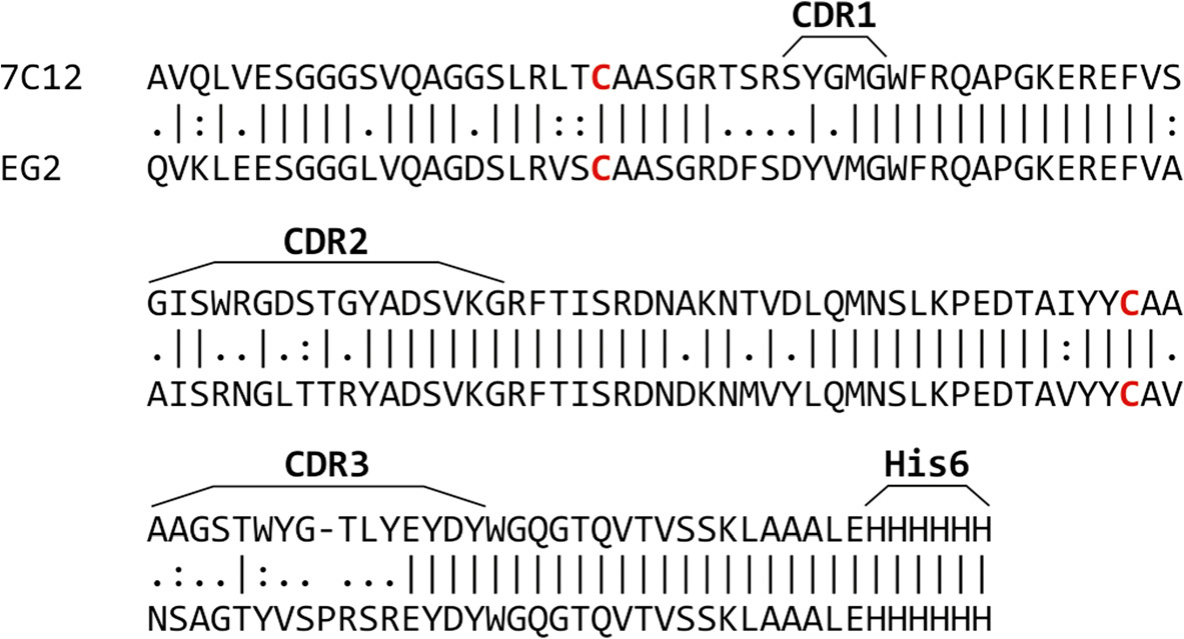
**Protein sequence alignment of investigated single-domain antibodies.** The amino acid sequences of sdAbs 7C12 and EG2 were aligned using the EMBOSS Pairwise Alignment Algorithms. The two cysteine residues forming an intradomain disulfide bridge are highlighted in red.

The codon-optimized coding sequences of EGFR-specific sdAbs 7C12 and EG2 originating from the published amino acid sequences were cloned into the *E. coli* expression plasmid pET-28b for cytoplasmic localization of the recombinant proteins. In both cases, the sequence encoding a hexahistidine epitope was translationally fused to the carboxyterminal region of the sdAbs to facilitate further purification, radiolabeling and immunological detection. A start codon for the initial methionine was introduced by the *Nco*I restriction site used for cloning. The sdAb coding sequences were under control of the T7 promoter and their expression was induced using IPTG. The putative proteins contain two cysteine residues forming an intradomain disulfide bridge [34], as highlighted in Figure 1. Protein sequence alignment revealed a sequence similarity of both sdAbs of 80.4% and differences by several amino acid substitutions in each complementary determining region (CDR), which are involved in antigen binding [35].

### Cultivation characteristics in different media

During the cultivation of *E. coli* SHuffle® T7 Express harboring the recombinant constructs pET-28b:7C12 or pET-28b:EG2 in Luria-Bertani (LB) broth, Terrific broth (TB) or EnPresso medium, respectively, growth characteristics and medium pH conditions were recorded and summarized in Table 1. Bacterial cultures grown in EnPresso medium showed higher induction and final cell densities compared to LB and TB media. With respect to buffer capacities, the EnPresso medium was capable of maintaining pH between 6.5 and 7.0 throughout the cultivation, while in LB and TB media pH increased above 8 during prolonged cultivation. However, independently from the different cultivation media, cell densities of the strain harboring pET28b:EG2 were somewhat higher than those of the strain carrying pET-28b:7C12.

**Table 1:** **Growth and pH characteristics of different *E. coli *** **cultures**

**Strain**	**Medium**	**OD**_**600**_**at induction**	**pH at induction**	**OD**_**600**_**at harvest**	**pH at harvest**	**CDW (g/L) at harvest**
SHuffle® T7 Express [pET-28b:7C12]	LB	1.0	7.3	1.4	8.8	0.38
	TB	1.1	7.2	8.3	8.4	2.24
	EnPresso	10.6	6.5	13.1	6.9	3.54
SHuffle® T7 Express [pET-28b:EG2]	LB	1.0	7.3	1.8	8.7	0.49
	TB	1.0	7.1	10.7	8.1	2.89
	EnPresso	10.2	6.6	19.8	7.0	5.35

Samples from each cultivation were taken at the time point of induction and harvest and subsequently, OD_600_ and pH were determined. Cell dry weight (CDW) was calculated at the end of cultivation, whereas one unit of OD_600_ corresponds to a dry cell weight of 0.27 g/L [52].

**Figure 2:**
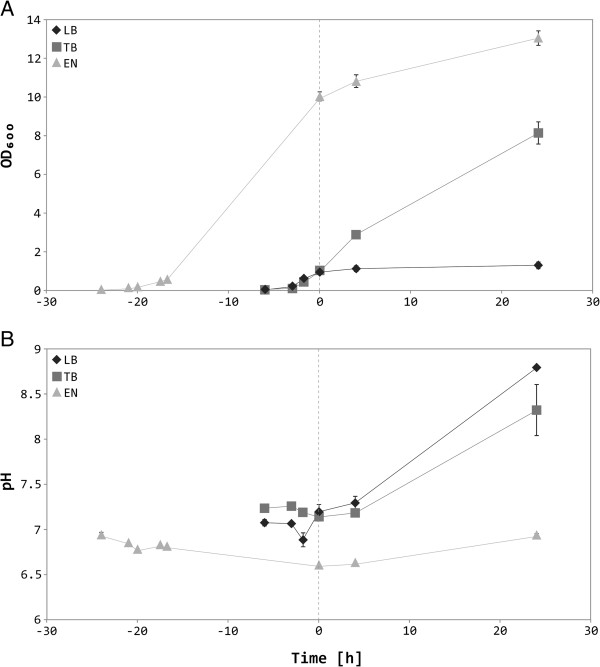
**Recorded data during cultivation of recombinant *E. coli *strain SHuffle® T7 Express [pET-28b:7C12] in different culture media.** Cell density **(A)** and pH **(B)** measurements for EnPresso medium (EN), Luria-Bertani medium (LB) and Terrific Broth (TB). The time point of induction (1 mM IPTG) is highlighted with a dashed vertical line. Cultures in LB and TB were grown for 6 h at 30°C before induction, whereas EnPresso cultures were grown for 24 h at 30°C before induction. All cultures were harvested 24 h after induction. The cultivation experiment was repeated twice and average values are shown.

Figure 2 presents the detailed cell density and pH data for the strain transformed with pET-28b:7C12. The use of different cultivation media had remarkable influence on the cell density of the culture, whereas the EnPresso medium was superior to the other media. The final OD_600_ at 24 h after induction was ~13 in EnPresso medium, whereas in LB and TB cultures the final OD_600_ at the time point of harvest were ~1.4 and ~8.3, respectively.

### Recombinant protein production

**Figure 3:**
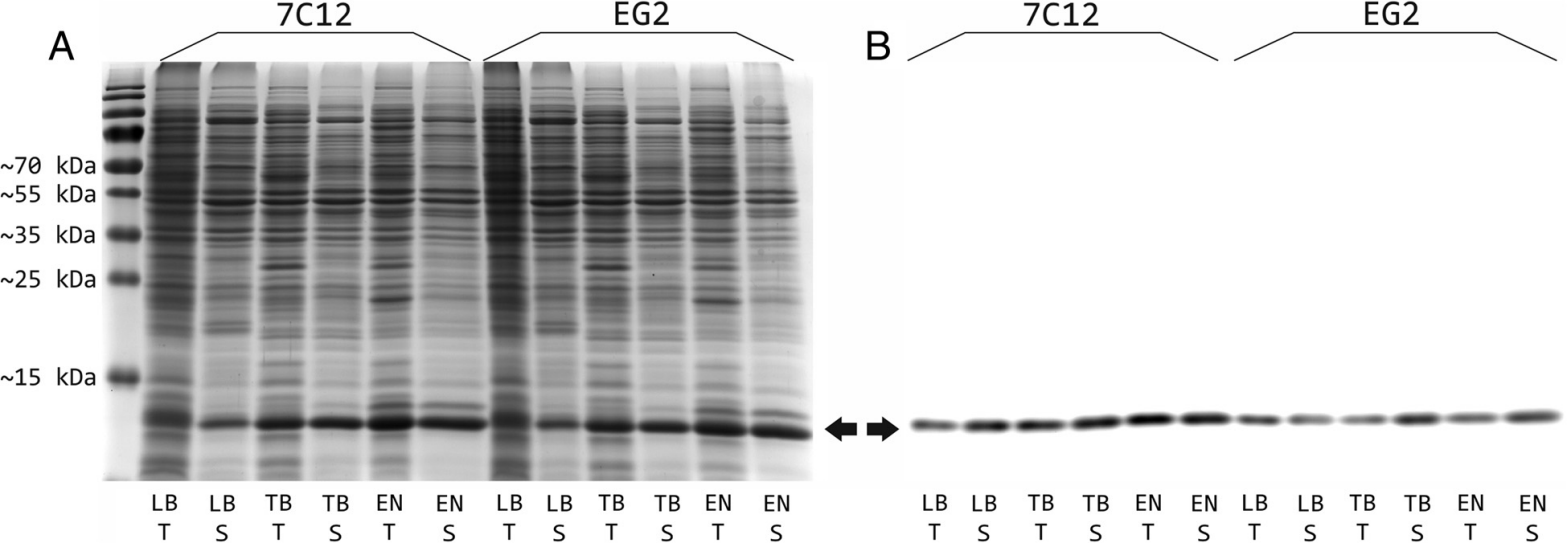
**Recombinant protein production of recombinant *E. coli *strains SHuffle® T7 Express [pET-28b:7C12] and [pET-28b:EG2] using different cultivation media.** Total and soluble protein fractions of recombinant strains at 24 h after induction separated by SDS-PAGE with subsequent Coomassie staining **(A)** or immunological detection using an HRP-conjugated Penta His antibody directed against the hexahistidine epitope **(B)**. In order to allow direct comparison between different cultivation media, all samples were diluted depending to their optical density to equal cell concentration before cell lysis and gel loading. LB = Luria-Bertani broth, TB = Terrific broth, EN = EnPresso medium, T = total cell lysate, S = soluble fraction of cell lysate. The experiments were repeated twice and representative pictures are shown.

Total and soluble protein fractions of recombinant strains at 24 h after induction separated by SDS-PAGE are visualized in Figure 3A. For the strains *E. coli* SHuffle® T7 Express [pET-28b:7C12] and SHuffle® T7 Express [pET-28b:EG2] cultured in the three different media, similar protein productivities were observed. In all of these cultures most of the expressed sdAbs were in the soluble form as suggested by the virtually equal sizes of total and soluble sdAb bands.

Immunological detection using an antibody directed against the carboxyterminal hexahistidine epitope of the recombinant proteins led to a single band confirming the results of the SDS-PAGE (Figure 3B).

### Purification of the recombinant single-domain antibodies

**Figure 4:**
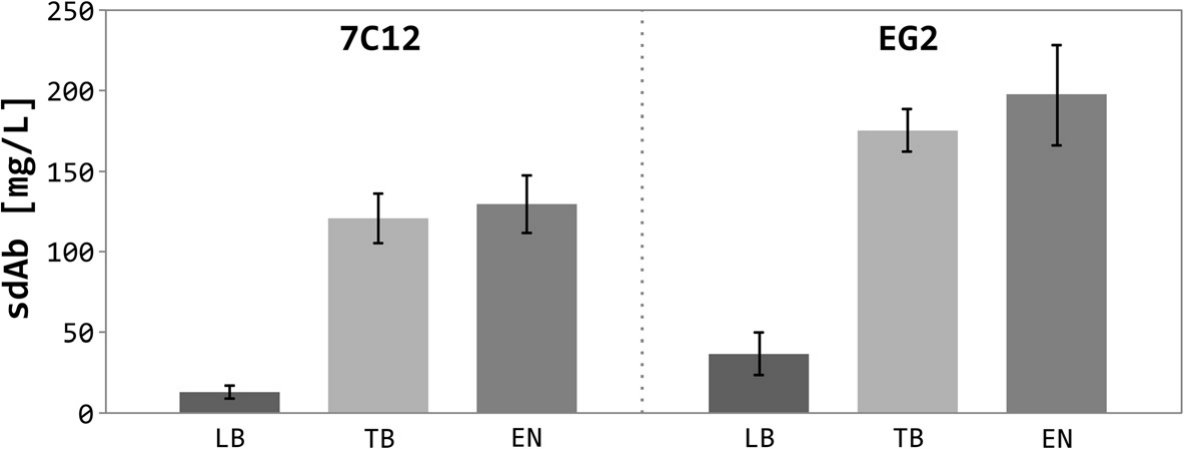
**Production yield of sdAbs in *E. coli *SHuffle® T7 Express [pET-28b:7C12] and [pET-28b:EG2].** Comparison of protein production yield obtained for expressed sdAbs. All cultures were grown in triplicate and the reported values correspond to the average.

In order to quantify the amount of cytoplasmically expressed sdAb in different media, 50 mL of each culture were harvested at 24 h after induction, purified by immobilized metal affinity chromatography (IMAC), and the amount of purified protein was determined colorimetrically. Although cultivation of *E. coli* SHuffle® T7 Express in EnPresso media resulted in the highest cell density and calculated cellular dry weight at the time point of harvest, no significant differences in the amount of expressed sdAb per liter culture were observed between EnPresso media and TB (Figure 4). However, the amount of purified recombinant protein differed between the studied sdAbs. For 7C12, on average, the protein productivity is ~120 mg/L and ~130 mg/L for TB and EnPresso media, respectively, whereas ~13 mg/L recombinant sdAb where achieved for LB broth. The average productivity of EG2 was higher in each investigated culture medium compared to 7C12, ranging from 37 mg/L for LB broth to ~175 mg/L and ~200 mg/L for TB and EnPresso media, respectively.

For the standard protein expression strain *E. coli* BL21(DE3) cultivated in EnPresso media, the recombinant protein productivity was slightly higher compared to *E. coli* SHuffle® T7 Express. An amount of ~220 mg EG2 and ~140 mg 7C12 could be purified from 1 L of EnPresso media, respectively, representing an approximately 10% higher production yield.

### Analysis of free sulfhydryl and disulfide groups

Since the intradomain disulfide bridge is critical for the stability and functionality of the sdAb molecule, we quantified the fraction of oxidized cysteine residues in the purified proteins expressed either in the cytoplasm of *E. coli* SHuffle® T7 Express or in *E. coli* BL21(DE3). As described above, the former represents an engineered strain to promote disulfide bond formation in the cytoplasm, whereas the latter is a standard strain for protein production.

The results summarized in Table 2 show that most of the sdAbs from EnPresso cultures are present as oxidized proteins after expression in the cytoplasm of *E. coli* SHuffle® T7 Express and purification. Only ~80% of the sdAbs exist as oxidized proteins after purification, if *E. coli* BL21(DE3) is used as expression host. Thermal unfolding of sdAbs expressed in different *E. coli* strains revealed, that lack of a disulfide bonds is consistent with lower thermal stability. As shown in Table 2, the melting temperatures of sdAbs expressed in SHuffle® T7 Express are increased compared to identical proteins generated in BL21(DE3).

**Table 2:** Quantification of oxidized cysteine residues in purified sdAbs and corresponding melting temperatures

**Protein**	**Strain**	**Medium**	**% oxidized cysteine residues**	***T***_**m**_**[°C]**
7C12	SHuffle® T7 Express	EnPresso	96.1 ± 0.6	64.8 ± 0.8
	BL21 (DE3)	EnPresso	84.7 ± 1.1	61.0 ± 1.0
EG2	SHuffle® T7 Express	EnPresso	97.6 ± 0.5	60.5 ± 0.5
	BL21 (DE3)	EnPresso	81.2 ± 0.7	58.9 ± 0.7

Purified sdAbs from EnPresso cultures were denatured and incubated with (S_red_) or without (S_ox_) tris(2-carboxyethyl)phosphine. After removal of the reducing agent by acetone precipitation, proteins were incubated with Ellman’s reagent. The formation of 2-nitro-5-thiobenzoate was quantified in a spectrophotometer by measuring the absorbance at 412 nm (A_412nm_), and the percentage of oxidized cysteine residues was calculated using the formula: 100% - 100 * A_412nm_[S_ox_]/A_412nm_[S_red_]. Thermal unfolding was assessed at pH 7.4 by measuring the absorbance at 280 nm as a function of temperature.

**Figure 5:**
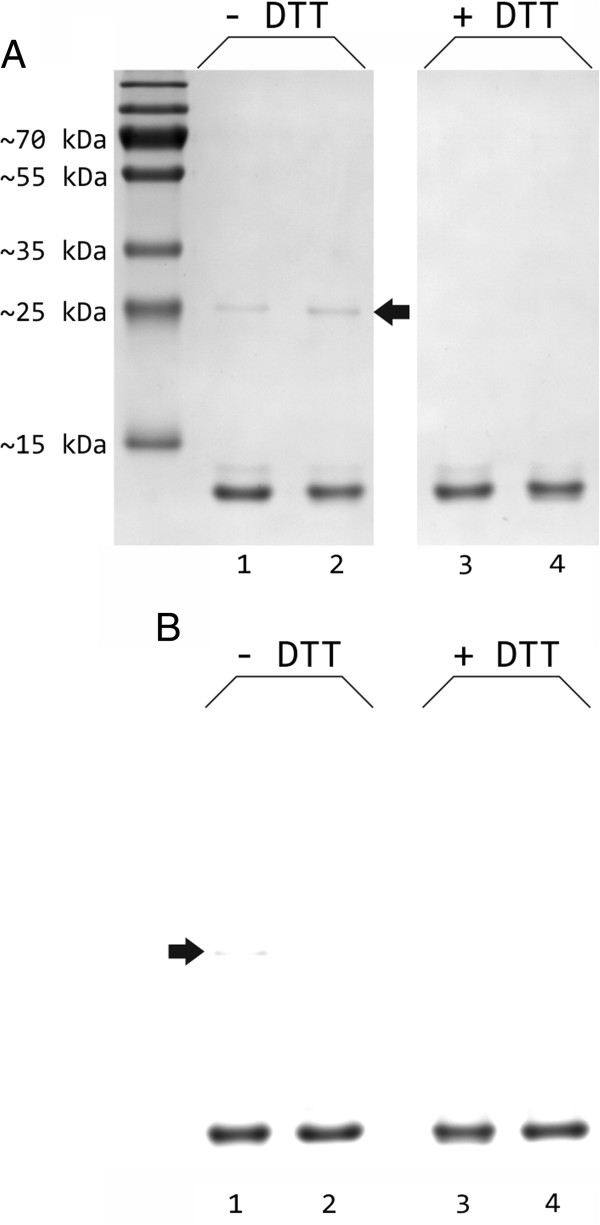
**Analysis of purified sdAbs under reducing and non-reducing conditions.** Ten μg of purified sdAbs 7C12 (lane 1 and 3) and EG2 (lane 2 and 4) generated in *E. coli* SHuffle® T7 Express were analyzed by SDS-PAGE in the absence and presence of the reducing agent dithiothreitol (DTT) with subsequent Coomassie staining **(A)** or immunological detection using a HRP-conjugated Penta · His antibody directed against the hexahistidine epitope **(B)**. A weak band corresponding to sdAb dimer is highlighted by an arrow. SDS-PAGE and immunostaining were repeated twice and representative pictures are shown.

Ten μg of purified sdAbs expressed in *E. coli* SHuffle® T7 Express were analyzed further by SDS-PAGE with or without prior reduction, and subsequently transferred onto a PVDF membrane for Western Blot analysis using an antibody directed against the carboxyterminal hexahistidine epitope (Figure 5). Colloidal Coomassie and immunostaining of unreduced sdAbs revealed the existence of a marginal amount of dimers in the size range of ~25 kDa. These results prove the oxidation of thiol groups and the predominant formation of an intradomain disulfide bridge, if the sdAbs are expressed in the cytoplasm of *E. coli* SHuffle® T7 Express.

### Radiolabeling and cell binding studies

**Figure 6:**
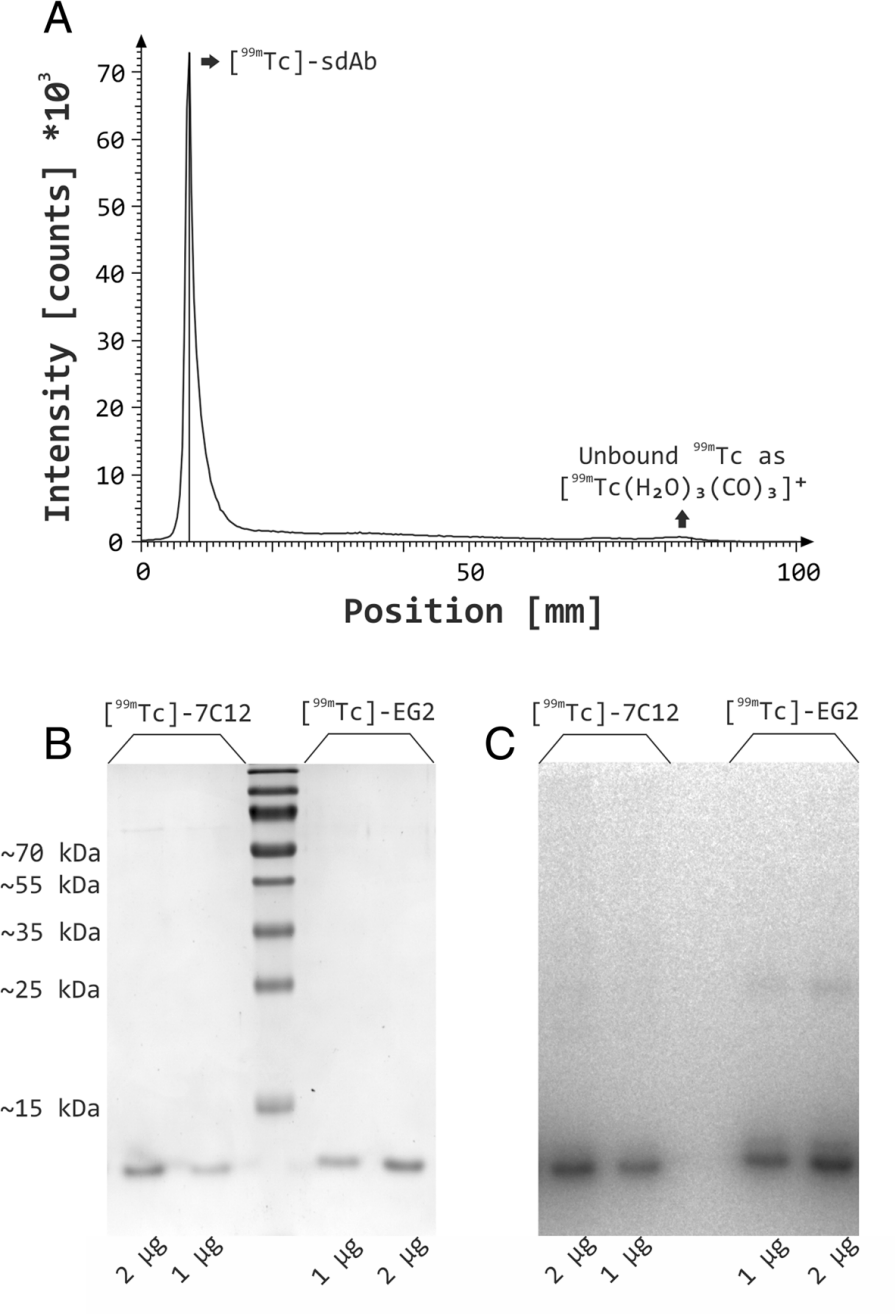
**Radio-TLC, SDS-PAGE and autoradiography analysis of**^**99m **^**Tc-labeled sdAbs.** After radiolabeling of 7C12 at its carboxyterminal hexahistidine epitope with [^99m^Tc(H_2_O)_3_(CO)_3_]^+^, the radiochemical yield was determined by radio-TLC **(A)**. Identical graphs were obtained for EG2 and are therefore not shown. ^99m^Tc-sdAbs were further analyzed by SDS-PAGE **(B)** and autoradiography **(C)** in the absence of DTT. The protein contents were visualized by Coomassie staining. Radiolabeling, SDS-PAGE and autoradiography analysis were repeated twice and representative pictures are shown.

Purified sdAbs from *E. coli* SHuffle® T7 Express were radiolabeled by incubating [^99m^Tc(H_2_O)_3_(CO)_3_]^+^ with the proteins (100 μg; 1 μg/μL) at 37°C for up to 60 min. Within this time period, a radiochemical yield of >98% (as analyzed by radio-TLC) was obtained and longer incubation times did not improve the radiochemical yield (Figure 6A). Radiolabeled proteins were analyzed by SDS-PAGE (Figure 6B) and autoradiography (Figure 6C) in the absence of dithiothreitol. A single predominant protein species corresponding to the monomeric radiolabeled sdAb is clearly visible in the Coomassie stained gels as well as in the autoradiographic images.

**Figure 7:**
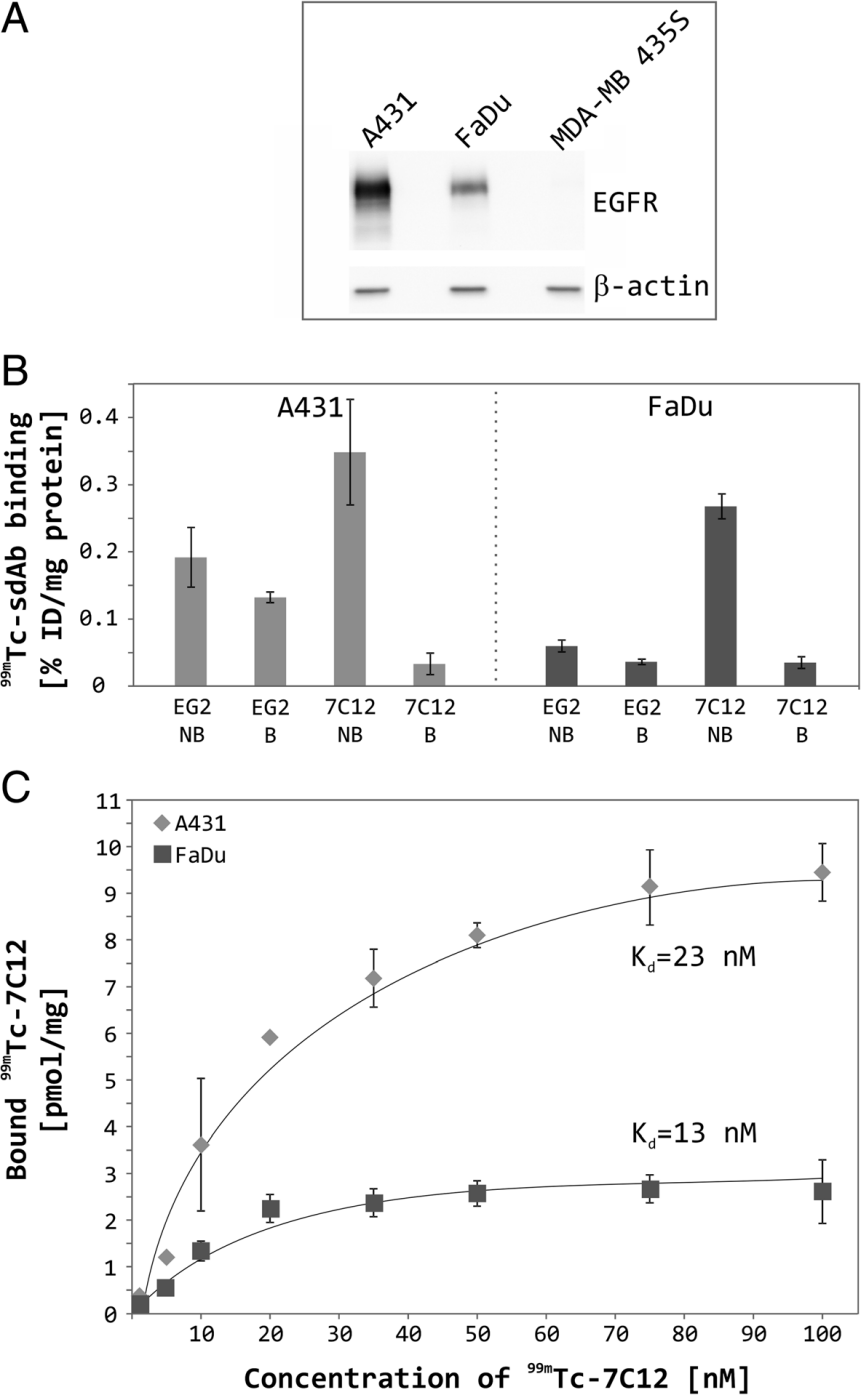
**Analysis of EGFR expression levels and binding affinities of sdAbs to human EGFR-presenting cells.** Whole-cell lysates of exponentially growing cells (A431, FaDu, MDA-MB 435S) were prepared and equal amounts of total cellular proteins were separated by SDS-PAGE on 10% polyacrylamide gels. After Western Blot transfer onto PVDF membranes, EGFR and β-actin proteins were detected by incubation with the respective specific antibodies followed by HRP-coupled antibodies and chemiluminescence detection **(A)**. *In vitro* specificity of ^99m^Tc-7C12 and ^99m^Tc-EG2 on A431 and FaDu cells was investigated after 1 h incubation on ice **(B)**. Binding of radiolabeled sdAbs was blocked by 40-fold excess of unlabeled Cetuximab. Binding data is expressed as percent of injected dose per mg protein (% ID/mg protein). NB = non-blocked, B = blocked. For *in vitro* binding studies, two dimensional cultures of A431, FaDu and MDA-MB 435S cells were incubated with increasing concentrations of ^99m^Tc-7C12 **(C)**. Total binding was measured in the absence of and nonspecific binding in the presence of 1 mM unlabeled sdAb. Specific binding was calculated as the difference between total and nonspecific binding. Binding studies were repeated twice and representative saturation curves for the EGFR-positive cell lines A431 and FaDu are shown. For the EGFR-negative cell line MDA-MB 435S, no specific binding was observed.

In order to evaluate the binding specificity and affinity of cytoplasmically expressed sdAbs to human EGFR, two dimensional cell cultures of epidermoid carcinoma (A431), squamous carcinoma (FaDu) and ductal carcinoma (MDA-MB 435S) cells were incubated with radiolabeled sdAbs. These tumor cell lines present different expression levels of the receptor on their cell surface as determined by quantitative immunostaining (Figure 7A). EGFR abundance is significantly elevated in A431 cells and moderate in FaDu cells, whereas MDA-MB 435S cells contain no detectable amount of the receptor.

To gain insight into the epitope specificity of investigated sdAbs, EGFR was blocked with a 40-fold excess of unlabeled Cetuximab before adding radiolabeled sdAbs (Figure 7B). Binding of ^99m^Tc-7C12 with A431 and FaDu cells is EGFR-specific as it could be blocked substantially by Cetuximab. However, ^99m^Tc-EG2 binding was only slightly reduced by pre-incubation of cells with Cetuximab. This observation indicates that ^99m^Tc-EG2 recognizes an epitope not overlapping with that of Cetuximab. Based on these results, ^99m^Tc-7C12 was selected for further analysis of EGFR binding. In Figure 7C, the saturation binding curves of ^99m^Tc-7C12 for the EGFR-positive cell lines A431 and FaDu are shown. Scatchard analysis were applied to calculate a K_d_ of 23 ± 5 nM and B_max_ of 16 pmol/mg protein for A431 and a K_d_ of 13 ± 4 nM and B_max_ of 3 pmol/mg protein for FaDu cells, respectively. For the EGFR-negative cell line MDA-MB 435S, no specific binding could be observed.

## Discussion

As the vast majority of antibodies, single-domain antibodies (sdAbs) contain cysteine residues forming disulfide bonds, whose presence is crucial for the stability and functionality of the sdAb molecules. Hence, the correct formation of the S-S bond and the associated proper folding of the soluble sdAb monomers appear to be at least as important for their application in the fields of tumor targeting and drug delivery as their high yield production. Bacterial cysteine oxidation occurs predominantly after a protein has been secreted from the highly reducing environment of the cytoplasm into the more oxidizing periplasm, requiring a bacterial leader peptide. However, this strategy often suffers from low yields of active protein due to poor translocation across the cytoplasmic membrane as well as aggregation in the periplasm [36]. In order to address these problems, genetically engineered double mutant strains defective in both, the thioredoxin and glutathione pathways, have been developed [37,38]. These mutations render the cytoplasm more oxidizing, allowing the formation of structural disulfide bonds in this compartment. Furthermore, the constitutive cytoplasmic expression of the disulfide bond isomerase DsbC facilitates the correct folding of cysteine containing proteins [33,39]. Herein, we compared the efficiency of protein production for two EGFR-specific sdAbs in the cytoplasm of the commercially available *trxB gor* mutant strain *E. coli* SHuffle® T7 Express between three different cultivation media. In addition to Luria-Bertani (LB) and Terrific broth (TB), we used an enzyme-based glucose release system (EnPresso medium) for cytoplasmic expression of sdAbs. As observed earlier by Krause and colleagues [40], considerably higher cell densities are obtained with the EnPresso medium compared to LB and TB (Figure 2). Most of the recombinant cytoplasmic proteins are produced in the soluble fraction. Surprisingly, the cytoplasmic productivity per cell of total and especially soluble protein was similar between the different media, but due to substantially higher cell densities, TB and EnPresso media outperform LB. Comparative purification of cytoplasmically expressed sdAb originating from the complete biomass of a 50-mL culture resulted in 100–200 mg/L soluble sdAb for TB and EnPresso media and five to ten times less recombinant protein for LB media (Figure 4). This is in excellent agreement with published expression data with microbial protein productivity of 100 mg/L for *E. coli*[41-43] and up to 100 mg/L for *Saccharomyces cerevisiae*[23,44,45]. Differences in protein productivity between 7C12 and EG2 suggest that the overall production yield is strongly depended on the antibody of interest [46]. The standard protein expression strain *E. coli* BL21(DE3) shows slightly higher production levels compared to the *trxB gor* mutant strain *E. coli* SHuffle® T7 Express. This observation might be explained by the additional constitutive expression of the disulfide bond isomerase DsbC in the cytoplasm of the latter strain, which limits the readily available protein production machinery for recombinant proteins.

As described for sdAbs, functional formats of conventional antibodies such as antigen binding fragments (Fabs) and single-chain variable regions (scFvs) can be expressed using *E. coli* either in the cytoplasm or in the periplasm [46-48]. Intracellular overexpression of antibody fragments often results in insoluble aggregates (inclusion bodies) and periplasmic expression generally suffers from low yields. The use of mutant strains that promote disulfide bond formation in the cytoplasm also represents for these antibody derivatives a successful and economic strategy to produce recombinant antibodies with protein productivities of up to 30 mg/L [49-51].

As shown here, there are no substantial differences in the protein productivity of *E. coli* cultures grown in rather simple media such as TB or 2YT compared to complex high cell density cultivation media. However, the former media do not feature the pH maintenance capacity of EnPresso media, which is of special importance for long term and economic large scale production as carried out in industrial applications.

Higher expression levels of correctly folded recombinant antibody fragments may be achieved by modifying the culture conditions such as temperature and oxygen transfer. For example, Ukkonen and co-workers were able to increase the cell density as well as the active yield of a recombinant enzyme expressed in *E. coli* by the combined use of EnPresso medium and high-aeration shake flasks [52].

Characterizations of the cytoplasmically expressed and purified sdAbs demonstrate that the vast majority of the cysteine residues are in the oxidized state and almost no sdAb dimers are detectable (Table 2 and Figure 5). These results indicate that the desired intradomain S-S bond within the sdAb monomer is formed favorably over an aberrant intermolecular disulfide between two sdAb molecules. This is most likely attributable to the rather slow kinetics of protein oxidation in the cytoplasm as already discussed by Bessette and co-workers for the oxidation of alkaline phosphatase in the cytoplasm of a comparable *E. coli* strain. According to the authors, at slow oxidation rates, the native conformation of the recombinant protein determines the disulfide bond formation and leads to bridging of the proper cysteine residues [33]. Interestingly, a substantial fraction of oxidized sdAbs expressed in the reducing cytoplasm of the standard expression host *E. coli* BL21(DE3) could be detected. Similar findings were already described in literature, *e.g.* Järviluoma and colleagues purified comparable amounts of functional sdAb derivative from *E. coli* BL21(DE3) and from *trxB gor* mutant Origami(DE3) cells [53]. This could be due to the fact that several thiol groups are oxidized spontaneously during purification procedure by atmospheric oxygen [54]. However, random oxidation of proteins possessing more than one S-S bond results in scrambled disulfides, which may require complicated rearrangement. The decreased amount of oxidized sdAbs originating from BL21(DE3) cultures is consistent with the lower thermal stability (Table 2). Since disulfide bonds increase the conformational stability of proteins considerably [55], sdAbs with free thiol groups unfold at lower temperature. This underlines the importance of correct S-S bond formation and the associated proper folding of the soluble sdAb monomers.

In order to proof the functionality of the expressed sdAbs, we radiolabeled the purified proteins with ^99m^Tc and investigated specific binding to human EGFR-expressing tumor cells (Figures 6 and 7). The expressed sdAbs recognize non-overlapping epitopes of the receptor, since ^99m^Tc-7C12 competes for binding to EGFR with the whole antibody Cetuximab and ^99m^Tc-EG2 does not. The reported affinities of ^99m^Tc-7C12 of 2.3 nM [17] and 3.7 nM [19] differ from that we obtained here. The main reason therefore might be the method of measurement. Gainkam and colleagues determined the affinity of 7C12 either to soluble recombinant EGFR by surface plasmon resonance [17] or to A431 cells in PBS [19], whereas here a binding assay to full-length EGFR presented by living cells in the presence of serum was applied. Since *in vivo* serum proteins [56], in particular endogenous ligands of the targeted receptor (*e.g.* epidermal growth factor (EGF), betacellulin, heparin-binding EGF-like growth factor, and transforming growth factor-α) compete with the sdAbs for binding to EGFR, the obtained affinities are supposed to be closer to *in vivo* reality. The surprising variety in the affinity of the sdAbs between A431 and FaDu cells can be explained by the different cellular context as it was observed similarly for EGF recently [57].

## Conclusions

Here we demonstrated, for the first time, the combined use of high cell density cultivation media with a genetically engineered *E. coli* mutant strain designed for the cytoplasmic formation of proper disulfide bonds for the production of up to 200 mg/L functional soluble single-domain antibodies (sdAbs). The ability to produce substantial amounts of correctly folded recombinant sdAbs as well as tailor-made multispecific and multivalent derivatives thereof in *E. coli* paves the way for their application in the fields of tumor targeting, molecular imaging and drug delivery. In addition, the promising cultivation and expression conditions used in this study could be adopted for high-yield production of other more complex proteins with multiple disulfides as well.

## Methods

### *E. coli* strains and plasmids

*Escherichia coli* NEB 5-alpha (*fhuA2* Δ*(argF-lacZ)U169 phoA glnV44* Φ*80*Δ *(lacZ)M15 gyrA96 recA1 relA1 endA1 thi-1 hsdR17*) was used in molecular cloning experiments, whereas *E. coli* SHuffle® T7 Express (*fhuA2 lacZ::T7 gene1* [lon] *ompT ahpC gal* λ*att:*:pNEB3-r1-*cDsbC* (Spec^R^, *lacI*^*q*^) Δ*trxB sulA11 R(mcr-73::miniTn10*--Tet^S^)2 [dcm] *R(zgb-210::Tn10* --Tet^S^) *endA1* Δ*gor ∆(mcrC-mrr)114::IS10*) and *E. coli* BL21(DE3) (*fhuA2* [lon] *ompT gal (*λ *DE3)* [dcm] *∆hsdS*) were used for expression of the recombinant sdAb. All strains were purchase from New England Biolabs. The plasmid pET-28b (Merck KGaA) coding a kanamycin resistance was used for cytoplasmic protein production.

### Bacterial media

In this study, three different media were used for the cultivations: (i) Luria-Bertani broth (LB), (ii) Terrific broth (TB) and (iii) EnPresso medium (EN). Luria-Bertani broth contained (per liter): 10 g tryptone, 5 g yeast extract and 5 g sodium chloride. Terrific broth contained (per liter): 12 g tryptone, 24 g yeast extract, 4 mL glycerol, 100 mL 0.17 M KH_2_PO_4_, and 100 mL 0.72 M K_2_HPO_4_. EnPresso medium (BioSilta) was prepared as described by the manufacturer [40]. Briefly, in a 1 L shaking flask, four EnPresso® medium tablets were added to 100 mL of sterile water. Before inoculation, the glucose releasing enzyme was added to a final concentration of 0.3 U/L. Before induction of protein expression with IPTG, the addition of two EnPresso booster tablets results in a higher concentration of complex nutrients [52].

All media were supplemented with 50 μg/mL kanamycin sulphate for maintaining the selective pressure.

### Cell culture

For binding and uptake studies, three different adherent human tumor cell lines were used: the epidermoid carcinoma cell line A431 (ATCC® Number: CRL-1555), the squamous cell carcinoma cell line FaDu (ATCC® Number: HTB-43), the ductal carcinoma cell line MDA-MB 435S (ATCC® Number: HTB-129). All cells were cultured in T25 or T75 cell culture flasks in 6 or 12 mL DMEM plus 10% heat-inactivated fetal calf serum (FCS), respectively, and incubated in a humidified atmosphere of 95% air/5% CO_2_ at 37°C. For harvesting, counting and sub-cultivation, culture media was removed and cells were washed twice with PBS. After the addition of 1% trypsin-EDTA, cells were incubated for 5 min at 37°C in order to detach from the bottom of the culture flask. Then, DMEM plus 10% FCS was added and cell suspension was transferred into 50-mL conical centrifuge tubes and spun down for 5 min at 150 × g and room temperature. The cell pellet was resuspended in 10 mL DMEM plus 10% FCS and the cell number as well as viability was determined using a CASY cell counter (Roche Diagnostics) according to the manufacture’s protocol. All cell lines were confirmed to be mycoplasma negative using the LookOut mycoplasma PCR detection kit (Sigma-Aldrich) and were tested monthly.

### Sequence analysis

For *in silico* reverse translation of amino acid sequences the Sequence Manipulation Suite was used [58]. Physical and chemical parameters of sdAbs were calculated with the ProtParam tool [59]. The EMBOSS Pairwise Alignment Algorithms were used for pairwise alignments of protein sequences [60].

### Molecular cloning

The sdAb coding sequences were commercially synthesized including a 5′ restriction site for *Nco*I and a 3′ restriction site for *Hin*dIII, respectively. The ~380-nt DNA fragments were digested with appropriate restriction endonucleases and ligated into *Nco*I/*Hin*dIII-linearized pET-28b plasmid. The ligation reactions were transformed into chemically competent *E. coli* NEB 5-alpha cells. The DNA sequences of the resulting recombinant constructs pET-28b:7C12 and pET-28b:EG2 were checked by automated DNA sequencing.

### Cultivation and expression of recombinant proteins

Freshly transformed *E. coli* SHuffle® T7 Express and *E. coli* BL21(DE3) harboring the recombinant plasmids pET-28b:7C12 or pET-28b:EG2 were inoculated in 5 mL of LB broth containing 50 μg/mL of kanamycin and cultivated at 30°C for overnight in an orbital shaker with 50 mm offset and shaking speed of 200 rpm. After that, 1 mL of this pre-culture was inoculated into 100 mL EnPresso medium in 1000 mL conical glass flasks and grown at 30°C. After overnight cultivation, two EnPresso booster tablets and an additional dose of the glucose releasing enzyme (0.6 U/L) were added to each 100 mL culture. At the same time, recombinant protein expression was induced by the addition of 1 mM IPTG. Cultivation was continued as described above for 24 h.

For comparison purposes, recombinant protein production in standard LB and TB broth, respectively, was tested under similar cultivation conditions as described for the EnPresso technology. Therefore, 1 mL of the identical pre-culture as used for the EnPresso cultivation was inoculated into 100 mL LB or TB in 1000 mL conical glass flasks and grown at 30°C until the optical density at 600 nm reached ~1. Then, IPTG was added to the culture broth to a final concentration of 1 mM and cultivation was continued for 24 h.

Cell growth was monitored by measurement of optical density in 1 mL cuvettes at 600 nm using a double-beam UV/Vis spectrophotometer SPECORD® 210 (Analytik Jena). For pH measurement, 1 mL samples were harvested by centrifugation and pH was determined from the supernatant with a micro pH electrode (Mettler-Toledo).

For final harvest, cultures were collected and chilled on ice for 5 min and centrifuged for 15 min at 6,000 × g and 4°C. After removal of the supernatant, cell pellets were either stored at -20°C or subjected to purification procedure immediately.

For analysis of recombinant protein yield, 1 mL samples were pelleted for 15 min at 6,000 × g and 4°C. Supernatants were discarded and the pellets were frozen at -20°C. After thawing on ice, pellets were resuspended in xTractor cell lysis buffer (Clontech Laboratories) supplemented with endonuclease (Thermo Scientific Pierce) and incubated on ice for 15 min to lyze the cells. Samples were then centrifuged at 14,000 × g for 5 min to remove cell debris. Total proteins of the insoluble and soluble fraction from the lysates before centrifugation as well as soluble protein fractions from the lysate supernatant after removal of debris and insolubles were analyzed by SDS-PAGE.

### Purification of recombinant proteins

A high-capacity Ni-iminodiacetic acid (IDA) resin was used for purification of hexahistidine tagged recombinant sdAbs by immobilized metal affinity chromatography (IMAC). The gravity-flow-based chromatography was carried out under native conditions according to the manufacturer protocol (Clontech Laboratories). Efficient cell lysis was achieved by addition of 1 mL xTractor cell lysis buffer (Clontech Laboratories) supplemented with EDTA-free protease inhibitor cocktail (Roche Diagnostics) and 25 U endonuclease (Thermo Scientific Pierce) to 200 mg bacterial cell pellet. After incubation on ice for 15 min and centrifugation at 10,000 × g and 4°C for 20 min for removal of cellular debris, the clarified supernatant was loaded onto a gravity-flow column containing 1 mL of prepacked resin and incubated at room temperature for 30 min. Before elution of the sdAbs by addition of elution buffer containing 300 mM imidazole, the column was washed twice with increasing imidazole concentrations of 20 and 40 mM. Removal of imidazole and buffer exchange was achieved by dialysis against PBS using a cellulose ester membrane with a molecular weight cut-off of 3.5-5 kDa (Spectrum® Laboratories).

### Gel electrophoresis and Western Blot analysis

Denaturing sodium dodecyl sulfate-polyacrylamide gel electrophoresis (SDS-PAGE) was carried out according to a standard protocol [61]. For each gel, PageRuler Plus Prestained Protein Ladder (Thermo Fisher Scientific) was used as molecular weight ladder standard. After electrophoresis, proteins were transferred onto a PVDF membrane (Merck KGaA), subjected to radioluminography or stained with PageBlue protein staining solution (Thermo Fisher Scientific) according to the manufacturer’s instructions.

For Western Blot analysis, PVDF membranes were probed with horseradish peroxidase (HRP)-conjugated Penta · His antibody (Qiagen) directed against the hexahistidine epitope of sdAbs. The primary leporine monoclonal EGF Receptor (D38B1) XP® and β-actin (13E5) antibodies (Cell Signaling Technology), respectively, and a goat anti-rabbit HRP-conjugated antibody (Sigma-Aldrich) were used for immunostaining of human EGFR and β-actin.

Detection of bound conjugates was performed with the ECL Prime Western Blotting detection reagent (GE Healthcare) in combination with the STELLA imaging system (Raytest). *In silico* quantitative analysis of average band intensities were performed with the Advanced Image Data Analysis (AIDA) program (Raytest).

### Protein determination

Protein concentration was determined colorimetrically with the *DC* Protein Assay (Bio-Rad Laboratories) according to the manufacture’s microplate assay protocol using bovine serum albumin as protein standard.

### Estimation of free sulfhydryl and disulfide groups

The fraction of oxidized cysteine residues in the native sdAb samples was determined as described recently [48] with slight modifications. Briefly, 50 μg of purified protein expressed in EnPresso medium were mixed with 300 μL denaturation buffer composed of PBS with 4 M urea and split into two 150 μL aliquots (S_red_ and S_ox_). After the S_red_ aliquot was reduced by the addition of 10 mM tris(2-carboxyethyl)phosphine (TCEP), both samples were incubated for 1 h at room temperature. In order to remove the reducing agent, proteins of both samples were precipitated by acetone. Therefore, five volumes of cold (-20°C) acetone were added and mixed. After incubation for 30 min at -20°C, the samples were centrifuged for 5 min at 15,000 × g and the supernatant was carefully removed. Both pellets were washed with 90% cold acetone, air dried, resuspended in 120 μL denaturation buffer containing 1 mM 5,5′-Dithiobis(2-nitrobenzoic acid) and incubated at room temperature for 5 min. The absorbance of both samples was measured photometrical at a wavelength of 412 nm, and the percentage of oxidized cysteine residues was calculated using the formula:

$$100\%–100*A_{412\mathrm{nm}}\left[S_{\mathrm{ox}}\right]/A_{412\mathrm{nm}}\left[S_{\text{red}}\right]$$

### Thermal unfolding

Thermal unfolding was followed by UV absorbance spectroscopy at 280 nm using a double-beam UV/Vis spectrophotometer SPECORD® 210 (Analytik Jena) equipped with a Peltier cooled cell holder [62]. Temperature was increased by 1°C increments at a rate of 1°C/min. A cuvette with a 1 cm path length was used, and the protein concentration was 500 μg/mL in 50 mM potassium phosphate buffer, 150 mM NaCl, pH 7.4.

### Radiolabeling of purified sdAbs

Purified sdAbs were labeled at their carboxy-terminal hexahistidine epitope with ^99m^Tc-tricarbonyl intermediate [^99m^Tc(H_2_O)_3_(CO)_3_]^+^ at 37°C as described elsewhere [63,64]. The labeling process of the sdAbs (R_f_ = 0) was monitored by radio-TLC using ITLC-SA plates (Merck Millipore) in combination with a mobile phase of methanol and 1% concentrated HCl. As control, separate radio-TLC analyses of [^99m^Tc(H_2_O)_3_(CO)_3_]^+^ (R_f_ = 1) and ^99m^TcO_4_^-^ (R_f_ = 0) were performed in the same mobile phase. Evaluation of radio-TLC was carried out using a radioactivity thin layer analyzer (Rita Star, Raytest).

If necessary, unbound [^99m^Tc(H_2_O)_3_(CO)_3_]^+^ was separated from the labeled sdAbs by spin filtration using 0.5 mL centrifugal filters with a molecular weight cut-off of 3 kDa (Merck Millipore).

Radiolabeling of the sdAbs was confirmed by SDS-PAGE followed by electronic autoradiography of the gel using a radioluminography laser scanner (Raytest).

### *In vitro* binding studies of sdAbs

Cells were plated in 24 well cell culture microplates (Greiner Bio-One) at a density of 5*10^4^ cells/0.5 mL/well (n = 4) and incubated for 48 h prior to addition of radiolabeled sdAbs. After 48 h, cells were pre-incubated for 30 min at 4°C before the addition of different concentrations of radiolabeled sdAbs ranging from 1 nM to 100 nM. The cell culture microplates were further incubated for 60 min at 4°C. Following treatment with radiolabeled sdAbs, cells were washed three times with PBS in order to ensure removal of loosely attached proteins from the cellular membrane. Finally, cell lysis was achieved by the addition of 1% SDS/0.1 M NaOH and incubation for 30 min at room temperature with vigorous shaking. The radioactivity in the cell extracts was quantified using an automated gamma counter (PerkinElmer Life and Analytical Sciences). Total protein concentration in cell extracts was determined as described above.

## Competing interests

The authors declare that they have no competing interests.

## Authors’ contributions

KZ designed and conducted all experiments and prepared the manuscript. SW, FK and KZ carried out the cultivation and expression experiments as well as protein purification, characterization, radiolabeling, and cell binding studies. CF and HS supervised the radiolabeling experiments and participated in whose data analysis and interpretation. SW participated in the design of experiments as well as data analysis and interpretation. HS contributed to design and coordination of the study and participated in drafting of the manuscript. All the authors read and approved the final manuscript.
